# Supplementary material for: Are carbon emissions trading and green financial instruments synergistic? -Comprehensive quantitative research based on content analysis
Source: PLoS One. 2024 Mar 7;19(3):e0298601. doi: 10.1371/journal.pone.0298601 (PMC10919601; doi:10.1371/journal.pone.0298601)
Supplement: S1 Data — (ZIP) [file pone.0298601.s001.zip › Data/地级市Z_F工作报告.zip!/║■▒▒/╗╞╕╘╩╨/└┤╫╘╣1⁄2╓┌║┼í╛┬φ┐╦╩2╛▌═°í┐_2018.docx]

各位代表：

　　现在，我代表市人民政府向大会报告工作，请予审议，并请各位政协委员和其他列席人员提出意见。

　　一、2017年工作回顾

　　过去一年，在市委的坚强领导下，全市上下深入学习贯彻习近平总书记系列重要讲话精神，全面贯彻落实党中央、国务院和省委、省政府的各项决策部署，围绕“建设区域性增长极”的战略定位和“加快振兴崛起、决胜全面小康”的战略目标，突出“双强双兴”，推进“四大行动”，开拓创新，克难奋进，实现了本届政府良好开局。

　　经济保持平稳增长。预计全市实现地区生产总值1890亿元，增长7.2%;规模以上工业增加值增长8.5%;固定资产投资2205亿元，增长8%;一般公共预算收入133.33亿元，增长11.6%;社会消费品零售总额1081亿元，增长11%;外贸出口66200万美元，增长10%;城乡居民人均可支配收入分别达到26900元和12100元，增长8.5%、9.2%。全口径财政收入突破300亿元，国税总收入、地税总收入双双突破百亿大关;麻城、武穴、红安地方税收收入超过10亿元;全市税收过亿元企业达到8家、过5000万元企业达到23家。

　　转型升级步伐加快。三次产业结构调整为21.67：38.54：39.79。工业提档升级。黄冈高新区获批国家级高新区，“一区七园”共享金字招牌。市区产业项目取得重大突破，黄冈晨鸣、绿宇环保、星晖汽车等3个百亿工业项目开工建设，司米橱柜等重点项目加快推进，索菲亚家居等产业项目建成投产。全市规模以上工业企业1496家，高新技术企业215家，高新技术产业增加值占GDP比重达9.25%。新能源新材料、智能制造、新能源汽车、节能环保等新兴产业快速发展。农业提质增效。建成高标准农田73万亩，稻田综合种养达到80万亩，粮经饲统筹突破50万亩，建成各类现代农业示范园区221个，产业化经营组织过万家。农业产业化龙头企业达到320家，实现农产品加工产值1155亿元。有效期内“三品一标”品牌526个，国家地理标志保护产品73个。现代服务业活力增强。全域旅游全面推进，游客数量突破3000万人次，旅游总收入突破200亿元。获评央视首批全国“十佳魅力城市”，大别山世界地质公园创建接受联合国教科文组织评估，英山四季花海旅居小镇、团风一字水文化小镇、黄梅禅文化旅游区等加快推进。电子商务年交易额达到700亿元，村级电商服务站超过2000家。“五大幸福”产业快速成长，新经济迅猛发展。

　　改革红利加速释放。深化供给侧结构性改革，完成“地条钢”整治等去产能任务，全市商品房去化周期控制在合理区间，挂牌上市企业达到411家、累计直接融资47亿元，降低企业税费成本16亿元，谋划补短板十大工程项目20类、总投资1461亿元。改革试点有序实施，智慧城市、安全生产领域改革、义务教育教师县管校聘改革等49项国家级试点和龙感湖国有农场改革等24项省级试点进展顺利，小池荣获省首届改革奖。重点领域改革进程加快，司法责任制改革、城管执法体制改革、价格机制改革、国有林场改革、供销社改革、国资国企改革、统计改革等全面推进，市区公交改革全面完成，市农村综合产权交易中心基本建成。深化放管服改革，推进“互联网+政务服务”，实施行政审批“集成服务”，调整收费清单、市级中介服务事项清单，公布市县乡三级权力清单。在全省率先实现“34证合一”。

　　四大行动有力实施。推进招商引资一号工程，创新资本招商、产业招商，新引进亿元以上重大项目118个，新开工项目346个，新投产项目240个。全市456个亿元项目完成投资771.62亿元;“双百”项目新开工100个，完成投资300亿元;16个省重点项目全部开工，提前完成省定投资任务。PPP项目入库198个、投资规模5512亿元，市区地下综合管廊等5个PPP项目开工建设。新增市校合作高校5所，引进“千人计划”专家项目2个、创新团队63个，新建校企研发平台41家，获批国家级星创天地3家，新增创业孵化平台8个，引进创业项目316个，转化科技成果70项。新发展市场主体6.5万户，五类市场主体达到48.53万户。

　　三大攻坚战成效显现。在全省率先打响三大攻坚战。严控金融风险和政府性债务风险，建立监测、预警与化解机制，守住了不发生区域性系统性风险底线。聚力脱贫攻坚，落实“六个精准”“五个一批”，有望实现红安脱贫摘帽、162个贫困村出列、175522人脱贫。我市“五位一体”产业扶贫、红安“4321”健康扶贫、团风爱心超市等经验在全国全省推广，刘锦秀获评2017年全国脱贫攻坚奖奉献奖，“神峰模式”入选全国产业扶贫十大典型案例。持续推进大气、水、土壤污染防治三大行动，打造“雷霆行动”升级版，坚决落实中央环保督察反馈问题整改，十大专项整治成效明显。持续开展“绿满黄冈”行动，全面推行河湖库长制，《黄冈市天然林保护管理办法》出台实施。农村生活垃圾治理全面推进，龙感湖率先通过省级验收。109个乡镇污水处理厂启动建设。

　　城乡建设统筹推进。城镇化率提高到45.87%。市区城市“双修”有序实施，海绵城市、地下综合管廊加快建设，完成棚户区改造11805套，新开工市政工程36个、完成投资7亿元。新增公共绿地20.8万平方米，遗爱湖公园基本建成。共享单车亮相黄州。白潭湖片区大别山金融中心、市体育中心、市委党校迁建项目建成，实验小学新校区投入使用。城市管理步入全省先进行列，成功创建国家卫生城市，启动创建全国文明城市。黄团浠区域协同发展规划编制完成，三台河大桥、团黄大道、黄上公路等交通一体化项目先行启动。县城建设步伐加快，广济时光景区、蕲春工贸新天地等成为城市新亮点。城乡基础设施不断完善，合安九高铁黄梅段、黄黄高铁、江北铁路、“645”航道蕲春段等全面启动，沪蓉高速龟峰山支线、武穴长江大桥、棋盘洲长江大桥加快建设，完成“455”公路安防工程5860公里，黄州区获评全国“四好农村路”示范县。水利建设完成投资75.81亿元，白莲河水环境治理、小农水重点县工程、中小河流治理、外排能力建设、江河湖水系连通工程顺利实施。持续推进“宽带乡村”工程，行政村光纤通达率93%。电网建设投资17.85亿元，提前完成新一轮农网改造。临江四城、24个国家重点镇示范引领，特色小镇、美丽乡村建设全面推进。

　　民生质量持续改善。推进全民参保，养老保险、医疗保险等社会保险制度基本实现全覆盖，城乡医保整合、机关事业单位养老改革顺利推进。城镇新增就业85197人，26796人实现再就业。21.18万困难群众享受城乡低保，5.61万农村五保对象得到供养，2.34万人接受临时救助，19.6万贫困学生得到资助，11.2万残疾人获得“两项补贴”。新解决28.71万农村人口安全饮水问题，完成农村危房改造8295户，棚户区改造28498户。全面实行药品零差价，合理调整医疗服务价格，推进城市公立医院综合改革，推行医联体和分级诊疗制度，医改惠民效果彰显。推行互联网+计生+妇幼健康服务，计生服务管理水平提升。新建改建公办幼儿园33所，改善180所贫困村办学条件，实施义务教育“全面改薄”项目498个，城乡义务教育一体化、黄冈职教园五校整合运行等改革顺利推进，教育质量稳步提升。新建基层综合文化服务中心467个、村级文化广场489个。打造《大别山母亲》《槐花谣》《铁面金光悌审和珅》等一批文艺佳作，纪录片《苏东坡》央视播出，多部精品戏曲进京赴台演出。精神文明创建亮点纷呈，成功创建32个全国文明单位，黄冈中学获评第一届全国文明校园。安全生产形势总体平稳，食品药品监管水平不断提升。完善“1343”维稳机制、网格化管理和社会治安立体化防控体系，深化阳光信访、责任信访、法治信访，推进“七五”普法，平安黄冈、法治黄冈建设成效明显。罗田获全国综治最高奖“长安杯”。群众体育蓬勃开展，全民健身热潮兴起。公共财政支出八成以上投向民生领域，更多群众感受到发展变化，分享到发展成果，全面小康离我们越来越近!

　　自身建设不断加强。坚定不移把党的领导贯穿政府工作始终，巩固“三严三实”专题教育成果，扎实开展“两学一做”学习教育，与时俱进加强政府自身建设。认真落实全面从严治党主体责任，严格执行中央八项规定，旗帜鲜明反对腐败，坚决纠正损害群众利益的不正之风，严肃查处违纪违法案件。强化行政监察和审计监督，加强权力运行监督制约，政府系统党风廉政建设全面加强。自觉接受人大、政协监督，办理人大代表建议177件、政协委员提案387件。坚持依法行政，规范决策程序，提高执法能力，推进政务公开，法治政府建设走在全省前列。开通“12345”市民服务热线，开展政务大督查，深化行风评议，落实巡视反馈问题和百姓问政、行风热线曝光问题整改，政风行风持续好转。

　　国防动员、国家安全、人民防空、民兵预备役、优抚安置等工作进一步加强。与工会、共青团、妇联、工商联、科协等群团组织联系进一步密切。对台、对口援疆、民族宗教、外事侨务、移民、农垦、老龄、统计、保密、档案、气象、地震、方志、公积金管理、公共资源交易、新闻出版、广播电视、慈善事业、残疾人事业、妇女儿童事业、机关事务管理等工作都取得新的成绩。

　　奋斗历经艰辛，成绩来之不易。这是习近平新时代中国特色社会主义思想科学指引的成果，是党中央、国务院和省委、省政府坚强领导、关心支持的成果，是市委统揽全局、科学决策的成果，是市人大、市政协有力监督、大力支持的成果，是全市人民团结拼搏、共同努力的成果。在此，我代表市人民政府，向全市人民，向各位人大代表、政协委员，向各民主党派、工商联、无党派人士、人民团体、离退休老同志，向驻黄部队、武警官兵、政法干警、部省在黄单位，向所有关心支持黄冈发展的同志们、朋友们，致以崇高的敬意和衷心的感谢!

　　各位代表，我市正处在爬坡过坎、赶超发展阶段，发展不平衡的矛盾日益显现、不充分的问题比较突出，加快振兴崛起、决胜全面小康的任务十分艰巨。经济发展方面，总量不大、结构不优、质效不高的问题并存，传统产业转型升级步伐不快，战略性新兴产业发展滞后，现代化经济体系建设任重道远;项目支撑不足，实体经济困难不少，稳增长的压力较大;市区首位度不高、县域经济不强，工业化、城镇化短板明显。社会民生方面，脱贫攻坚任务繁重，财政收支矛盾突出，生态环保压力加大，基本公共服务供给能力不足，与人民群众对美好生活的期待还有很大差距。政府自身建设方面，一些政府工作人员履职能力不足、担当精神不强，庸政懒政怠政和“四风”问题仍然存在，发展环境有待进一步优化，政府效能有待进一步提高。我们将直面问题，变压力为动力，化挑战为机遇，在战胜困难中把握主动，赢得未来!

　　二、2018年工作安排

　　今年是全面贯彻落实党的十九大精神的开局之年，是改革开放40周年，是决胜全面小康、实施“十三五”规划承前启后的关键一年。新的一年，我市工业化、城镇化加速上升的阶段性特征更加鲜明，政策利好的叠加效应更加明显，区域战略地位更加突出，处于诸多机遇窗口期、干事创业黄金期，我们将坚定信心，努力开辟黄冈革命老区振兴崛起的新境界。

　　2018年政府工作的指导思想是：全面贯彻党的十九大精神，以习近平新时代中国特色社会主义思想为指导，认真落实省委、省政府和市委决策部署，坚持稳中求进工作总基调，坚持新发展理念，紧扣社会主要矛盾变化，按照高质量发展的要求，统筹推进“五位一体”总体布局，协调推进“四个全面”战略布局，坚持以供给侧结构性改革为主线，统筹推进稳增长、促改革、调结构、惠民生、防风险各项工作，大力推进改革开放创新，推动质量变革、效率变革、动力变革，打好防范化解重大风险、精准脱贫、污染防治的攻坚战，推进老区振兴崛起，决胜全面小康，为加快建设成为区域性增长极打下坚实基础。

　　主要预期目标是：地区生产总值增长8%左右，规模以上工业增加值增长8%以上，固定资产投资增长11%左右，社会消费品零售总额增长10%以上，一般公共预算收入增长8%以上，城乡居民人均可支配收入增长8%以上，居民消费价格指数涨幅控制在3.5%以内，城镇登记失业率控制在 4.5%以内，全面完成省下达的节能减排任务和环境质量改善目标。

　　发展为要，实干兴市。我们将把加快发展作为时代的最强音，用开放的思维谋划发展，用创新的理念引领发展，用改革的办法推动发展，加快构建现代化经济体系。把实干作为各级政府工作的标记，保持发展定力，一张蓝图绘到底，把业绩写在红色大地、印在群众心里!

　　围绕上述目标任务，重点做好八个方面的工作：

　　(一)推进高质量发展，构建现代产业新体系。深化供给侧结构性改革，一手抓传统产业改造升级，一手抓新经济培育壮大，加快新旧动能接续转换、经济结构持续优化。

　　提升传统产业。坚持工业主导，深入推进“工业倍增”计划，加快工业提档升级。实施千亿产业成长工程,制定重点产业振兴发展计划，改造提升传统产业，推动食品饮料、纺织服装、机械电子、汽车及零部件、建筑建材等迈向中高端，加快形成千亿产业。实施“万企万亿”技改工程, 推进祥云30万吨合成氨、索菲亚工业4.0生产线、马勒三环“机器换人”等一批重大技改项目，力争当年完成技改投资100亿元。开展智能工厂/数字化车间试点，培育中船重工等一批智能制造示范企业。实施隐形冠军培育工程,支持广济药业、稳健医疗、科峰传动等企业专注核心业务，着力技术创新，深耕细分市场，“专精特新”发展，培育一批领军企业和领先行业。支持山河集团向高端化的城市人居环境建设服务商升级。实施质量品牌提升工程,引导农产品加工企业强化质量管理，增品种、提品质、创品牌，培育中粮粮油、伊利乳业、馥雅食品、襄大农牧等一批产值过20亿元农业产业化龙头企业，建设黄梅关山、武穴火车站等一批产值过50亿元农产品加工园，形成优质粮油、特色畜禽、奶业、中药材等一批百亿特色产业集群。

　　发展新兴产业。着力培育新兴支柱产业。加快金风科技、鸿路装配式建筑示范园等项目建设，支持李时珍医药、智轩科技、光谷英山生物医药园等做大做强，继续引进一批产业链项目，发展新能源汽车、装备及智能制造、新能源新材料、节能环保、大健康等战略新兴产业。着力培育百亿企业。建立一企一策政策体系，完善点对点服务机制，推进黄冈晨鸣、绿宇环保、星晖汽车等重大项目建设，支持葛洲坝兴业等企业创新发展，培育一批年产值过百亿的“旗舰”企业。着力推进现代服务业发展三年行动计划。推进旅游资源和品牌整合，加强“十佳魅力城市”品牌营销，做精红色经典、生态休闲、乡村体验、中医养生等主题旅游。推进东坡文化旅游区、麻城龟峰山和红安红色5A景区创建，支持蕲春创建国家中医药健康旅游示范区，加快卓尔“五景区五小镇”、 李时珍文化旅游区、英山温泉度假旅游区、黄梅电影风情文化小镇、太白小镇建设。大力发展大型游乐、综合演艺等文化创意产业。推广移动互联网、云计算、大数据、物联网创新应用，加快“新一代云计算中心”建设，推进互联网+,促进多业融合、联动发展。大力发展服务外包，培育总部经济。

　　振兴实体经济。引导各类要素“脱虚向实”，建立大企业“直通车”制度，深化“六抓六促”服务企业行动，强化要素保障，破解实体经济发展难题，提振企业家信心。落实财政支持实体经济系列政策和创业扶持奖补政策，整治“红顶中介”，清理涉企收费，降低制度性交易成本、用能和物流成本。推进大别山金融工程升级，增强金融服务实体经济能力，加快金融改革创新，推进多层次资本市场建设，着力解决好“融资难、融资贵”问题。用好县域经济发展调度资金，做强政府性融资担保公司，设立大别山振兴发展产业基金、新兴产业投资基金和中小企业发展基金，全面组建转贷基金，搭建中小企业应急周转平台，助推实体经济振兴发展，让实体经济根深叶茂、四季长青!

　　(二)做强经济支撑，打造区域增长新引擎。挖掘增长潜力，做大经济总量，做优经济存量，做实经济增量，提升发展质量，构建“四梁八柱”，形成区域增长的坚实支撑。

　　突出园区主战场。掀起二次创业，加快黄冈高新区发展，着力将高新区打造为政策特区、人才特区、技术特区、资本特区，引领市域经济创新发展。拓展核心区发展空间，做实园区投融资平台，完善园区基础设施，提高承载能力。探索“一区七园”发展模式，促进协调发展、错位发展、特色发展。实行项目异地落户利益共享机制，发展“飞地经济”。深化与东湖高新区合作，推动共建光谷黄冈科技产业园。加快推进健帆生物、生产力促进中心、中交建PPP项目和在建百亿工业项目，促进黄冈产业园提质发展。支持广药生物产业园、蕲春健康新城、东湖高新红安产业园、麻城通航产业园、团风华夏幸福产业新城、黄梅龙源科技产业园、龙感湖纺织面料生产基地发展。加快推进“满园行动”，培育一批百亿园区。规范项目入园、建设、投产、运营及退出机制，提高投资强度、单位产出率和贡献率。开展园区闲置土地清理处置，优化资源配置。

　　突出项目主抓手。持续开展项目攻坚，打好老区牌、生态牌、扶贫牌、长江牌，包装策划一批大项目、好项目。做实项目前期，抓好“两库”建设，完善三年滚动投资计划，形成接续不断、滚动实施的良性循环。重点围绕大别山振兴发展、扶贫开发、长江经济带发展和补短板十大工程，加强项目对接、跟踪、落地。继续实行领导领衔项目计划，全力推进黄冈晨鸣等省“三类”、司米橱柜等市“三类”项目建设。加快推进合安九高铁黄梅段、黄黄高铁等重大项目，启动蕲太高速西段、麻竹高速延长线、G347巴河特大桥等重点工程，谋划推进巴河燕矶长江大桥、京九客专黄冈北段、江北高速、沪蓉高速至麻竹高速红安连接线、黄黄高铁沿线集疏运公路等项目。做好存量项目PPP转化，引进社会资本运营管理，推广EPC等模式。

　　突出消费主潜力。深入推进“十大扩消费行动”，挖掘消费潜能，推进消费升级，更好发挥消费对经济增长的基础性作用。加快构建现代流通体系，促进区域市场一体化。扩大服务消费、信息消费、线上线下互动消费，促进中高端产品消费，加快发展智能家居、健康养老、金融保险等新型业态，增加高质量产品的有效供给。改善消费环境，保护消费者权益，繁荣消费经济。

　　突出民营主力军。落实促进民营经济发展“18条”，构建“亲清”政商关系，创优环境，支持发展。全面实施市场准入负面清单制度，破除歧视性限制和各种隐性障碍。落实保护产权政策，依法保护民营企业和企业家的财产权、人身权，慎用人身强制、资金冻结及财产扣押措施。减少无关会议、活动对企业的干扰，对企业“无事不扰、有求必应”。建立领导干部联系重点民营企业和商会制度，健全民营经济发展联席会议制度，对反映的难点问题，实行台账管理、跟踪问效。民营经济是黄冈经济的顶梁柱，企业家是黄冈的宝贵财富。全社会要尊重创新创造、尊崇企业家精神，让企业家鲲鹏展翅九万里，逐梦扬帆再起航!

　　(三)深化改革开放，培育创新发展新动能。改革引领时代，开放赢得舞台，创新决定未来。要因时而变，随事而制，厚植创新发展新优势，塑造经济增长新动能。

　　以更大的力度深化改革。巩固和扩大“去降补”成果，提高供给体系质量。深化经济体制改革，完善产权制度和要素市场化配置。持续深化互联网+放管服改革，推进“一网覆盖、一次办好”。完善权力清单和责任清单制度，深化商事制度改革。加快信用体系建设，深化投融资体制改革，开展股权质押试点。推进国资管理体制、城管执法体制、应急管理体制、环保机构监测监察执法垂直管理制度改革，深化统计、价格、国有农场、供销社和国企改革，加快城发集团等政府融资平台市场化转型。推进中央和省级试点改革，扩大改革成果。鼓励基层创新，探索原创性改革，打造黄冈改革样本。

　　以更强的举措招商引资。坚持一号标准，落实一号任务，推进一号工程，力争新引进亿元项目300个以上，协议投资额2000亿元以上，资金到位率40%以上。注重产业招商，编制产业招商规划，探索设立重点产业投资基金，实行“一个产业、一名领导、一张招商图、一个团队、一个园区”工作机制，引进一批建链、强链、补链项目。注重平台招商，借助世界500强黄冈行、互联网+、市场中介等多种平台扩大招商。抢抓大型国有企业改革机遇，引进战略投资。注重以商招商，激励现有企业引进上下游配套企业和关联项目落户。注重友情亲情招商，做实创投基金，吸引更多的黄冈籍成功人士报效桑梓、回乡创业。发挥黄冈楚商、异地商会、黄冈校友、招商大使等资源优势，实现联动招商。建立招商和安商协同机制，提高注册率、开工率、投产率。

　　以更宽的视野扩大开放。力争外贸出口增长8%以上，实际利用外资增长15%以上。融入“一带一路”建设，大力推进更深层次更高水平双向开放。对接大武汉，主动寻求合作，承接功能疏解和产业转移。依托大长江，持续推进跨江协作，加快融入长江经济带和中游城市群。联通大别山，推动环大别山“五个一体化”合作。对标湖北自贸区，落实贸易和投资自由化便利化政策，积极申报新港保税区，推进口岸“大通关”体系和黄州一类水运口岸建设。推进团风港口产业园、大别山商贸物流园、红安传化振鑫物流园、大别山冷链物流等项目建设，做强开放平台。支持祥云集团、宏源药业等重点出口企业发展，推进船舶、生物医药、蕲艾、茶叶等出口基地建设，扩大出口规模，改善出口结构。办好“李时珍诞辰500周年”系列活动，扩大对外交流合作。我们将着力打造内陆开放新高地，让老区的开放度更高、竞争力更强，让黄冈发展的航船驶向更广阔的海洋!

　　以更浓的氛围创新创业。推进国家创新型城市建设，全面提升创新能力。加强创新主体培育，新增高新技术企业30家，新建企业科研平台30个，引进转化科技成果60项，R&D经费支出占GDP比重达到0.85%以上，高新技术产业增加值占GDP比重达到9.4%以上。推进人才发展体制机制改革，深入实施大别山英才计划和“黄冈工匠”培养行动，加强产业工人队伍建设。深化市校合作，引进实习实训大学生1.2万人，引进创业项目200个，出孵项目20个、入园项目10个。支持返乡创业、能人创业、草根创业，让一切想创业的人有机会、能创新的人有平台，点燃黄冈激情，汇聚成振兴崛起的磅礴力量!

　　(四)实施乡村振兴战略，建设富裕美丽新农村。农村大地是充满希望的热土，农村工作是大有作为的舞台。大力推进农业现代化建设，促进产业兴旺、生态宜居、乡风文明、治理有效、生活富裕。

　　让农业更强。坚持质量兴农、绿色兴农，提高农业规模化、科技化、信息化、标准化、品牌化水平。落实粮食安全行政首长责任制，加快推进粮安工程建设。完成76万亩高标准农田建设。推进互联网+现代农业，大力发展农村电商、农户微店，推动一二三产业融合发展。培育定制农业、创意农业、观光农业等新业态，发展民宿度假、养生养老、农耕体验等“美丽经济”。大力实施“三乡”工程，吸引市民下乡带活农民，吸引能人回乡激活农业，吸引企业兴乡盘活农村。培育新型农业经营主体和服务主体，发展现代农业产业化联合体、农民合作社联合社等规模经营。支持地标产品产业化发展，积极推进蕲艾、罗田板栗、英山云雾茶、“红安苕”、麻城福白菊、黄梅禅茶等“五进一出”。启动区域公共品牌创建工程，打造一批黄冈农产品公用品牌。加快农地确权颁证信息化建设，完善承包地“三权”分置制度，建成市县一体农村综合产权交易市场体系，使农地经营权活起来。金融支持农业发展，全面开展农村集体产权制度改革试点，盘活农村集体资产。探索组建农业投资平台公司，引导省农业担保公司向县市延伸，助推现代农业发展。

　　让农村更美。加强农村基础设施建设，推进外排能力建设、主要支流和中小河流治理、病险水库除险加固、河湖水系连通、西隔堤整体加固等工程建设。推进普通公路补短板工程，全面完成“455”公路安防工程，继续实施农村公路和危桥改造工程，加快通达规模以上自然垸、扶贫搬迁点和产业基地道路建设。支持浠水、蕲春、英山创建省级“四好农村路”示范县。推进农村四类重点对象危房改造，解决9.29万人安全饮水问题。持续推进农村人居环境整治，实施城乡生活垃圾无害化处理三年行动、“厕所革命”三年攻坚行动、“扫帚行动”和精准灭荒工程三年计划，加强农业面源污染防治，推进秸秆、畜禽粪污等资源化利用。全面开展村庄规划编制，强化乡村规划管控，加强农村“两违”和环境卫生整治，保持村庄整洁，保留乡村记忆，保护田园风光。持续推进300个美丽乡村示范村创建，统筹推进“一带一片”美丽乡村建设，引领新农村建设提档升级。健全自治、法治、德治相结合的乡村治理体系。做好村居两委换届选举工作，推进农村社区建设。培育“乡田同井，出入相友，守望相助”的乡风文明，让农民群众参与其中，得到美的享受、养成美的德行、过上美的生活!

　　让农民更富。全面推进农业农村“领头雁”计划，实施一村一名大学生计划和“乡土人才”计划，加强农业技术人才引进，鼓励高校毕业生、退伍军人、科技人员到农村施展才华，培养造就一支懂农业、爱农村、爱农民的“三农”工作队伍，带领群众创业致富。加强新型职业农民、互联网+现代农业科技精准化服务等职业技能培训，引导支持农民就业创业，挖掘经营性收入增长潜力，稳住工资性收入增长势头，释放财产性收入增长红利，拓展转移性收入增长空间，促进农民收入稳步增长，实现衣食无忧，走向生活富裕。我们相信，农民必将成为体面的职业，农业必将成为有奔头的产业，农村必将成为令人向往的美丽家园!

　　(五)坚持城乡一体，构筑融合发展新格局。统筹城乡规划、建设、管理三大环节，形成布局合理、特色鲜明、功能互补、联动发展的新型城镇化体系。

　　加快中心城市建设。市区要围绕建设区域性产业中心、创新中心、金融中心、现代物流中心、文化教育医疗中心的市域中心城市定位，增强承载功能、产业功能、带动功能。坚持市区一体，推进新一轮总体规划修编，探索“多规合一”。持续实施城市“双修”，推进海绵城市、地下综合管廊、江河湖水系连通、长河综合治理一期、城市防洪排涝等工程建设，修复城市生态。推进垃圾焚烧发电、生活垃圾处理场二期、三水厂扩建、停车场等工程建设，加快过街通道、城铁站配套、城市公交和智能交通体系建设，完善城市路网和市政基础设施，提升城市功能。启动赤壁公园环境治理，保护修复赤壁历史文化街区，全面建成并整体开放遗爱湖公园。规范物业管理，深入开展老旧小区、背街小巷综合整治。加快白潭湖片区发展，推进资本大厦、城市综合馆、亚行贷款城市环境综合整治项目建设，着力引进大健康、大金融、大文旅等产业项目，加快打造区域性商务服务集聚区。巩固提升城市创建成果，持续开展城管会战和市容环境、城市交通、违法建设等专项整治。积极推进黄团浠一体化发展，实施巴河至陶店、蕲州一级公路和团风红色旅游公路建设，探索推进公共服务一体化。对接湖北国际物流核心枢纽，谋划建设黄冈临空产业经济区。深化户籍制度改革，引导农村人口向城镇转移，帮助农村转移人口更好融入城市。

　　加快县城扩容提质。以城市总体规划修编为契机，支持县市城区空间拓展。抢抓棚改窗口期，加快老旧城区改造，加强市政设施建设和市容市貌综合整治，提升城市管理水平，打造宜居宜业县城。高位推进黄梅高铁新区、麻城西城新区、浠水一河两岸、蕲春城南新区、武穴城东生态新城和团风城区提档升级项目建设。积极推动文明城、卫生城、园林城、环保模范城创建，提升县城品质。加强分类指导，激励县市区在县域经济分类考核中争先进位，力争1-2个进入全省县域经济20强。

　　加快构建新型城镇体系。坚持全域规划，加快完善城乡规划体系，优化“一区两带”空间布局，加强城镇控制性详规编制，实现生产空间集约高效、生活空间宜居适度、生态空间山清水秀。完善小城镇功能，建设一批有影响力的交通枢纽型、旅游景观型、绿色产业型、商贸集散型和移民安置型小城镇，打造“四化同步”典范。提升镇域经济实力，发展一批财政收入过亿元的经济强镇和县域副中心。推进武穴城乡一体化试点，深化经济发达镇扩权试点，支持小池省级全面深化改革示范区建设，推进临江四城建设，支持七里坪镇国家级特色小镇建设，支持蕲州、九资河创建国家级特色小镇。

　　(六)聚焦绿色发展，树立生态文明新样板。牢固树立绿水青山就是金山银山的理念，像对待生命一样对待生态环境，把好山好水好风光永远留在黄冈大地，留给子孙后代!

　　涵养绿色生态。把长江大保护摆在压倒性位置，推进新一轮“雷霆行动”，坚决打赢污染防治攻坚战。抓好中央环保督察反馈问题整改，实现销号清零。开展第二次全国污染源普查，提高精准治污能力。坚持全民共治、源头防治，落实大气十条、水十条、土十条，打好蓝天碧水保卫战，确保完成省定目标。持续推进“绿满黄冈”，新造林面积30万亩以上。加大自然保护区、森林公园、湿地公园和地质公园保护力度。加快构建城乡连片、路河沿线、涵盖山水林田湖草的立体森林生态系统。继续推进白莲河生态保护与绿色发展示范区，湖北大别山、龙感湖国家级自然保护区建设。建立健全生态文明制度，加大生态环境保护和修复力度，完成生态保护红线划定工作，实施生态文明建设评价。落实河湖库长制，加强领导干部自然资源资产离任审计，健全环保督察长效机制，对一切环境违法行为零容忍。

　　发展绿色产业。严格环境准入和污染物总量排放控制，大力发展循环经济，建立企业环境信用与信贷等挂钩的激励机制，加快工业园区和传统产业循环式、绿色化改造，推进清洁生产。发展生态循环农业，推进田园综合体、“两清两减”示范基地建设。探索林业碳汇、生态效益补偿机制，创新生态资产抵押、收益权质押等融资方式，促进绿色金融产品创新和生态资产资本化，让生态资源成为老区人民的绿色银行。深入推进国土资源节约集约示范市创建。支持武穴矿产资源节约与高效示范工程试点、麻城国家循环经济试点市创建、罗田国家生态保护与建设示范区创建。

　　引领绿色风尚。倡导绿色消费，让自然、环保、节俭、健康的生活方式成为公众习惯和自觉行动。以创建大别山国家生态文明试验区为载体，广泛开展市县乡村四级生态文明创建，创建一批节约型机关、绿色社区、绿色工厂、绿色家庭。支持黄州、红安、罗田、武穴创建省级森林城市。

　　(七)下足绣花功夫，夺取脱贫攻坚新胜利。今年，要实现团风、罗田、英山3个贫困县摘帽，141个贫困村出列，163972人脱贫的目标。这是一场“啃骨头”的硬仗，要咬定脱贫目标，聚焦“准实”二字，确保重大政治任务如期完成。

　　坚持限期脱贫与质量标准相统一。保持定力，不急于求成，不搞数字脱贫、虚假脱贫。要静下心、沉下身、扎下根，用“搬石头”工作法，确保脱真贫、真脱贫。坚持时间服从质量，严格遵循“两不愁三保障”综合标准，既不降低标准，也不吊高胃口，保证现行标准下的脱贫质量。持续开展扶贫领域作风问题专项治理，加强扶贫领域资金监管，强化扶贫督查考核和责任追究。

　　坚持扶贫同扶志、扶智、扶能相结合。聚焦深度贫困地区、瞄准特殊贫困人口，精准帮扶，靶向发力。突出“五位一体”产业扶贫，坚持因地制宜，因户施策，建立稳步脱贫长效机制。落实健康扶贫政策，让贫困群众真正得实惠，减少因病致贫、因病返贫。妥善解决易地搬迁户与搬迁地的融合问题，确保搬得出、稳得住、能致富。强化政策兜底，统筹教育扶贫、金融扶贫、生态补偿等落地生根。高度重视扶志、扶智、扶能，加强政策引导，注重典型引路，激励贫困群众坚定信心，自力更生。加大技能培训和个性化帮扶，帮助贫困群众就业创业，激发脱贫内生动力。支持迅达药业、香江电器IPO上市。

　　坚持专项扶贫、行业扶贫、社会扶贫相衔接。落实“三个新增”的要求，加大项目资金整合力度，攻坚“三个深度”，确保贫困地区发展可持续、贫困对象稳定脱贫有门路。坚持政府主导、市场主体、社会参与，构建专项扶贫、行业扶贫、社会扶贫“三位一体”的大扶贫格局。

　　各位代表，众人拾柴火焰高。让我们携手同心，合力攻坚，全面小康路上决不漏掉一户、决不落下一人!

　　(八)着力改善民生，顺应人民群众新期待。坚持决策以民生为首选，支出以民生为首位，工作以民生为首要，让全市人民享有更多的获得感、幸福感、安全感。

　　打造文化强市。推进全国文明城市创建，实施文明实践“五大行动”，提高全社会文明程度。深化群众性精神文明创建活动，推进诚信建设和志愿者服务制度化。启动文化小康建设三年行动，抓好国家公共文化服务体系示范区建设，提高公共文化服务标准化、均等化水平。深入实施文化惠民工程，加快县市区“四馆两场”、乡镇综合文化站、村级服务中心建设，推进文化设施建设全覆盖。广泛开展全民阅读、百姓舞台、送戏下乡等群众性文化活动。完善扶持机制，实施文艺创作生产精品工程。加强历史文化遗产保护，争创国家历史文化名城。健全现代文化产业体系和市场体系，推进文化产业示范园区和示范基地建设，推动文化与科教、旅游等跨界融合，培育新型文化业态，引进培育一批有竞争力的文化市场主体。依托厚重人文，打造文化品牌，展现黄冈文化魅力。

　　优先发展教育事业。启动第三期学前教育三年行动计划，改善农村薄弱学校办学条件，加快市县城区教育资源增量扩容，推进义务教育优质均衡发展。启动高中阶段教育普及攻坚计划，推动适应新高考系列改革。深化职业教育产教融合、校企合作，推进现代学徒制改革试点。支持职业教育集团化发展，着手组建以黄冈职院为核心的职教集团。支持和规范民办教育发展。支持黄冈中学振兴发展、黄冈师院双一流建设。加强师德师风建设，培养高素质教师队伍，倡导全社会尊师重教。完善困难学生资助体系，着力解决中小学生课外负担重、“择校热”“大班额”等突出问题，办好人民满意的教育。

　　推进健康黄冈建设。实施健康黄冈“十大行动”，为人民群众提供全方位、全周期健康服务。坚持“三医联动”，深化医药卫生体制改革，发展医疗联合体，推进分级诊疗、家庭医生签约服务和健康管理，完善基层医疗卫生便民服务体系，加强全科医生、乡村医生队伍建设，继续解决好“看病难、看病贵”问题。深入开展爱国卫生运动，坚持预防为主，防控重大疾病。提升计生精细化服务管理水平。完成市中心医院、市妇幼保健院建设，实现市疾控中心整体迁建。传承发展中医药文化，推动“中医药健康谷”和中医药传承创新工程项目建设。支持社会办医，发展健康产业。构建养老、孝老、敬老政策体系和社会环境，推进医养结合，加快老龄事业和产业发展。加大国家“学生饮用奶计划”推广力度。推动全民健身和全民健康深度融合。举办黄冈马拉松、挺进大别山系列赛事，积极备战省运会，筹办市五运会。推进“双安双创”，落实“四个最严”，保障食品药品安全。

　　提高社会保障水平。坚持就业优先战略，完善积极就业政策，实现更高质量、更充分就业。统筹做好重点群体就业工作，加强困难群体就业帮扶。全面实施技能人才振兴计划，建立终身职业培训体系。构建和谐劳动关系，保证农民工工资支付。全面实施全民参保计划，实施统一的城乡居民基本医疗保险、大病保险和医疗救助制度，完善异地就医联网直接结算。加快建立多主体供给、多渠道保障、租购并举的住房制度，满足群众多层次居住需求。健全社会福利、慈善、优抚安置、法律援助等制度，健全农村留守儿童和妇女、老年人关爱服务体系，加强残疾人康复服务。完善最低生活保障制度，实现农村低保五保按标施保、应保尽保，努力让困难群众求助有门、受助及时，决不让一个困难群众生活过不去，决不让一个学生因家庭困难失学，决不让贫困代代相传!

　　创新社会治理。积极开展法治惠民、法治创建活动。加大全民普法力度，弘扬社会主义法治文化。落实矛盾纠纷市县乡村四级联排化解机制，拓展诉调结合和第三方参与社会矛盾化解的制度化渠道，加强社会心理服务，推进“律师进村、法律为民”。做好信访工作，维护人民群众合法权益和司法权威。深化平安黄冈创建，加强互联网安全管理，加快社会治安立体化防控体系建设，依法打击和惩治黄赌毒黑拐骗等违法犯罪活动，大力整治群众反映强烈的治安问题。加强“三社联动”，完善“网格化+”，推进管理集成、重心下移，实现政府治理和社会调节、居民自治良性互动。依法管理宗教事务，提高宗教工作法治化水平。深化安全生产领域改革，推进打非治违专项行动，守住安全生产红线。提升防灾减灾救灾能力，加强应急救援体系建设。

　　防控重大风险。开展互联网金融和其他非法集资风险排查，完善金融风险防控体系，确保不发生系统性风险。持续开展政府性债务清理，规范融资平台融资行为，加强政府债务风险评估和监测预警，打赢防范化解重大风险攻坚战。

　　深入开展国防教育和双拥共建，加强国防动员、民兵预备役和人民防空建设，支持国防和军队改革，加大人武部正规化建设力度。支持驻黄部队建设，巩固军政军民团结，推进军民融合深度发展。开展第四次全国经济普查，做好地震、档案、气象和方志等工作。

　　三、着力建设人民满意政府

　　新时代赋予新使命，新征程召唤新作为。我们将紧跟时代要求，努力打造勤政务实、廉洁高效、人民满意政府。

　　忠诚履职。牢固树立“四个意识”，坚决维护以习近平同志为核心的党中央权威和集中统一领导，坚决落实中央和省委、省政府的决策部署，自觉把政府工作置于市委的坚强领导之下，保证市委的决策部署在政府层面有力有效落实。巩固“两学一做”学习教育成果，认真开展“不忘初心、牢记使命”主题教育，坚定“四个自信”，始终把以人民为中心的价值追求融入血脉、铸入灵魂。

　　践行法治。把依法行政贯穿于政府工作的全过程，做到法定职责必须为、法无授权不可为，全面加强法治政府建设。依法接受人大及其常委会的法律监督和工作监督，自觉接受人民政协的民主监督，认真办理人大代表建议和政协委员提案。诚恳听取民主党派、工商联、无党派人士和人民团体的意见，主动接受监督。加强行政执法信息化平台建设，开展行政执法突出问题专项治理。健全决策机制，推进依法决策、科学决策、民主决策、高效决策。

　　务实担当。有多大担当才能干多大事业，尽多大责任才会有多大成就。 应该做的事，克服困难也要干;必须负的责，顶着压力也要担。不喊新口号、不标新立异。重大决策一以贯之，决不动摇折腾;重大项目一抓到底，决不半途而废;重大任务一鼓作气，决不无果而终。着力提高“五种思维”能力，提升“八种本领”，锻造担当的宽肩膀。健全正向激励机制，落实容错纠错机制，严格落实“三个区分开来”，为冲锋陷阵的闯将、攻城拔寨的猛将、有胆有识的干将撑腰壮胆，让我们的干部想干事、敢干事、干成事!

　　勤勉服务。服务是政府工作的本质。转职能、转方式、转作风，简政便民，优化服务流程、提高办事效率，改进服务方式、提升服务质量。强化效能问责和行政问责，大力整治“门好进、脸好看、事难办”“为了不出事，宁可不干事”等不作为、慢作为问题，大力倡导“立即办、马上办、我来办”“责任在我、执行在我、落实在我”。坚持绩效管理和责任管理，强化政务大督查，对法定责任、议定事项、既定目标，一追到底，见人见事见结果。

　　廉洁从政。认真落实全面从严治党主体责任，从严从实抓好政府系统管党治党。深入推进廉洁政府建设，健全资金管理、工程招投标、公共资源交易等制度，扎牢织密制度笼子。加强审计监督，完善权力运行制约和监督机制。全力支持纪委、监委工作，旗帜鲜明反对腐败。持之以恒正风肃纪，严格执行中央八项规定精神和省、市委实施办法，坚决纠正形式主义、官僚主义，坚决反对特权思想、特权现象，防止“四风”问题反弹回潮。

　　同志们，梦想照亮前方，奋斗赢得未来。让我们更加紧密地团结在以习近平同志为核心的党中央周围，在省委、省政府和市委的坚强领导下，凝心聚力，砥砺奋进，努力创造无愧于党、无愧于人民、无愧于时代的光辉业绩!
